# Twenty-year experience following aortic valve replacement in patients younger than 60 years of age

**DOI:** 10.1186/s13019-024-02776-x

**Published:** 2024-05-07

**Authors:** Yuki Imamura, Ryosuke Kowatari, Junichi Koizumi, Azuma Tabayashi, Daiki Saitoh, Hajime Kin

**Affiliations:** 1 Department of Cardiovascular Surgery, Iwate Medical University, 2-1-1 Idaidori, Yahaba-cho, Shiwa-gun, Iwate, Yahaba 028-3695 Japan; 2 Department of Thoracic and Cardiovascular Surgery, Hirosaki University, Hirosaki, Japan

**Keywords:** Aortic valve replacement, Younger patients, Long-term outcome, Mechanical valve

## Abstract

**Objective:**

Reports on long-term outcomes of surgical aortic valve replacement (AVR) for patients aged < 60 years are scarce in Japan. Hence, we aimed to evaluate these outcomes in patients aged < 60 years.

**Methods:**

Between March 2000 and December 2020, 1477 patients underwent aortic valve replacement. In total, 170 patients aged < 60 years who underwent aortic valve replacement were recruited. Patients aged < 18 years were excluded. Patient data collected from the operative records and follow-up assessments were reviewed.

**Results:**

The mean age was 49 ± 9 years, and 64.1% of patients were male. One-hundred-and-fifty-two patients (89.4%) underwent aortic valve replacement with a mechanical valve and 18 (10.6%) with a bioprosthetic valve. The mean follow-up period was 8.1 ± 5.5 years. No operative mortality occurred, and in-hospital mortality occurred in one patient (0.6%). Ten late deaths occurred, with seven cardiac-related deaths. The overall survival rate was 95.4 ± 1.7%, 93.9 ± 2.3%, 90.6 ± 3.9%, and 73.2 ± 11.8% at 5, 10, 15, and 20 years, respectively. Freedom from major bleeding was 96.4 ± 1.6% at 5, 10, and 15 years, and 89.0 ± 7.3% at 20 years. Freedom from thromboembolic events was 98.7 ± 1.3%, 97.3 ± 1.9%, 90.5 ± 4.5%, and 79.0 ± 11.3% at 5, 10, 15, and 20 years, respectively. Freedom from valve-related reoperation was 99.4 ± 0.6% at 5 years, 97.8 ± 1.7% at 10 and 15 years, and 63.9 ± 14.5% at 20 years.

**Conclusions:**

Patients aged < 60 years undergoing aortic valve replacement with a high mechanical valve implantation rate had favorable long-term outcomes.

**Supplementary Information:**

The online version contains supplementary material available at 10.1186/s13019-024-02776-x.

## Introduction

Surgical aortic valve replacement (SVAR) has long been the first-line treatment for aortic valve disease. The advent of transcatheter aortic valve replacement (TAVR) has expanded the treatment options. In addition to TAVR, minimally invasive cardiac surgery (MICS) has been increasingly performed in recent years [1]. Although TAVR was initially limited to elderly or high-risk patients, its indication has been expanded to low-risk or young patients [2–4]. More recently, valve-in-valve (ViV) TAVR has emerged as a treatment option for structural valve deterioration (SVD) [5]. With the increasing trend toward the use of bioprosthetic valves in Japan [6], it is important to evaluate their effectiveness and long-term outcomes. However, only a small number of studies have reported long-term results for patients aged < 60 years who underwent SAVR in Japan [7].

Therefore, this study aimed to evaluate the long-term outcomes of aortic valve replacement (AVR) for individuals aged < 60 years, predominantly using mechanical valves.

### Subjects

**Fig. 1:**
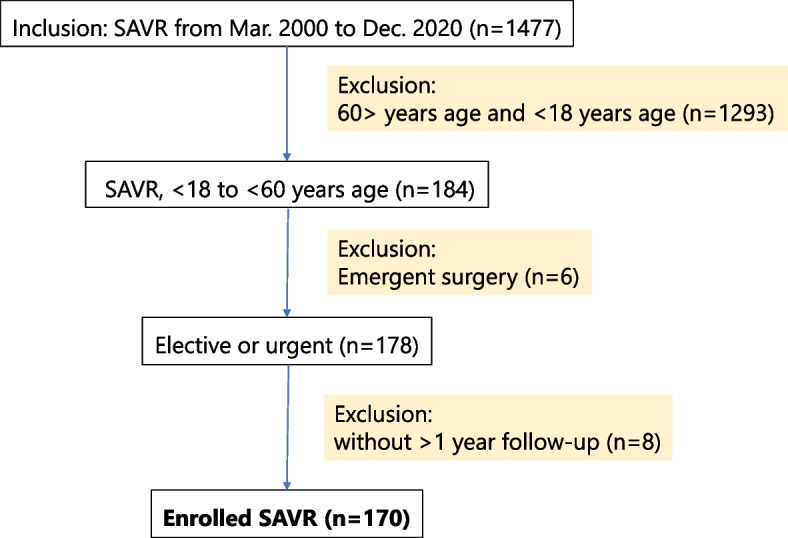
Study population and inclusion/exclusion criteria

A total of 1477 patients underwent AVR at our institution from March 2000 to December 2020. Patients were identified in the Iwate Medical University cardiac surgery databases. The inclusion criterion was AVR with a mechanical or bioprosthetic valve during the study period. We also included all patients undergoing concomitant procedures, including other valve procedures, aortic surgery, and coronary artery bypass grafting as well as patients who underwent AVR for infective endocarditis and aortitis. We excluded patients aged > 60 years and, all adolescents and young adults with congenital heart disease (age < 18 years) who underwent AVR, emergency cases and lost to follow-up within 1 year of their operation (Fig. 1).

## Methods

The primary endpoints were overall survival and cardiac-related mortality. The secondary endpoints were valve-related reoperation, major bleeding events, and thromboembolic events. Preoperative demographics, operative clinical characteristics, and postoperative in-hospital complications were obtained from operative notes. Long-term follow-up data were available for all patients by clinical visit, telephone, or written correspondence until a common closing date (April 2022). The mean follow-up period was 8.1 ± 5.5 years (range, 0.6–21.5 years).

This study was approved by the Iwate Medical University Institutional Review Board (MH2021-119). The requirement for individual informed patient consent was waived due to the retrospective nature of the study.

### Statistical analysis

Continuous normally distributed variables are presented as mean ± standard deviation. Non-normally distributed continuous variables are presented as median and interquartile range. Cumulative survival was estimated using the Kaplan–Meier method. Cox proportional hazards regression analysis was performed to estimate the hazard ratio for risk factors of late mortality. Statistical analysis was performed using the Statistical Package for Social Sciences, version 26.0, for Windows (SPSS, Chicago, IL, USA).

### Definitions of clinical outcomes

Operative mortality was defined as 30-day mortality and in-hospital mortality, including any deaths occurring after transfer to another hospital or long-term acute care facility [8]. The primary endpoints included overall survival, cardiac-related mortality, and reoperation. Sudden, unexplained death was considered cardiac-related mortality. The secondary endpoint was major adverse prosthesis-related events according to the guidelines for reporting mortality and morbidity following cardiac valve intervention [9].

## Results

### Patient characteristics

One-hundred-seventy patients were under the age of 60. Patient baseline and operative characteristics are summarized in Table 1; 64.1% of patients were male, and the mean age was 49 years (range, 24–59 years). Sixty-four patients (37.6%) were aged < 50 years and 25 (14.7%) were aged < 40 years. Aortic valve stenosis (*n* = 85, 50.0%) was the most frequent indication for surgery. The fundamental etiologies were as follows: bicuspid aortic valve (*n* = 74, 43.5%), degenerative (*n* = 51, 30.0%), infective endocarditis (*n* = 22, 12.9%), rheumatic (*n* = 17, 10.0%), and aortitis (*n* = 6, 3.5%).

**Table 1:** Preoperative characteristics of patients

Variables	*n* = 170
Age (y), mean (range)	49 (24–59)
Male sex, No. (%)	109 (64.1%)
Hypertension, No. (%)	52 (30.6%)
Hyperlipidemia, No. (%)	36 (21.1%)
Diabetes mellitus, No. (%)	13 (7.6%)
Chronic obstructive pulmonary disease, No. (%)	9 (5.3%)
Hemodialysis, No. (%)	4 (2.3%)
Smoking history, No. (%)	25 (14.7%)
Aortic stenosis, No. (%)	85 (50.0%)
Aortic insufficiency (severe), No. (%)	85 (50.0%)
AV disease etiology, No. (%)	
Degenerative	51 (30.0%)
Bicuspid	74 (43.5%)
Infective endocarditis	22 (12.9%)
Rheumatic	17 (10.0%)
Aortitis	6 (3.5%)
Surgical approach, No. (%)	
Median sternotomy	170 (100%)
Aortic valve replacement, No. (%)	
Mechanical prosthesis	152 (89.4%)
Biological prosthesis	18 (10.6%)
Concomitant procedures, No (%)	
Hemiarch aortic replacement	30 (17.6%)
Mitral valve plasty	14 (8.2%)
Mitral valve replacement	14 (8.2%)
Tricuspid valve plasty	9 (5.3%)
Coronary artery bypass grafting	8 (4.7%)
Cardiopulmonary bypass time (min) mean ± SD	140.3 ± 59.6
Ischemic time (min) mean ± SD	103.9 ± 44.8

*AV* aortic valve, *MICS* minimally invasive cardiac surgery, *SD* standard deviation

### Operative characteristics

A total of 170 patients underwent AVR, with 152 (89.4%) undergoing mechanical AVR and 18 (10.6%) receiving bioprosthetic AVR. The operative characteristics are listed in Table 1. The valve characteristics are listed in Online Resources 1 and 2. The mechanical valve types were ATS (Medtronic, Minneapolis, MN, USA) in 84 patients, On-X (On-X Life Technologies Inc., Austin, TX, USA) in 38 patients, St. Jude (St. Jude Medical Inc., St. Paul, MN, USA) in 23 patients, and CarboMedics (Sorin SpA, Milan, Italy) in 7 patients. The bioprosthetic valve types were INSPIRIS RESILIA (Edwards Lifesciences LLC) in 6 patients, Carpentier-Edwards Magna Ease (Edwards Lifesciences LLC, Irvine, CA, USA) in 10 patients, Mosaic bioprosthesis (Medtronic, Inc.) in 1 patient, and Trifecta (Abbott Vascular, Santa Clara, CA, USA) in 1 patient.

Regarding the surgical approach, all 170 patients underwent median sternotomy. The concomitant operations are listed in Table 2. Thirty patients (17.6%) required hemiarch aortic replacement and 28 (16.4%) underwent mitral valve surgery. The mean cardiopulmonary bypass time was 140.3 ± 59.6 min and mean ischemic time was 103.9 ± 44.8 min.

**Table 2:** Early outcomes

Variables	Overall (*n* = 170)
Operative mortality	1 (0.6%)
30-d mortality	0
Hospital mortality	1 (0.6%)
Perivalvular leak (> moderate)	1 (0.6%)
Stroke	0
Reoperation for bleeding	7 (4.1%)
Heart block	1 (0.6%)
Atrial fibrillation	27 (15.8%)
Mediastinitis	0

Values are presented as No. (%)

### Early outcomes

The early outcomes are summarized in Table 2. There was no operative mortality. The overall in-hospital mortality rate was 0.6% (*n* = 1). The patient with bicuspid valve died due to sepsis. Regarding postoperative complications, reoperation due to perivalvular leak was required in 1 patient (0.6%), and reoperation due to bleeding was required in 7 patients (4.1%). There was no incidence of stroke. Postoperative atrial fibrillation was reported in 27 patients (15.8%) and heart block in 1 patient (0.6%).

### Late outcomes

#### Survival

**Fig. 2:**
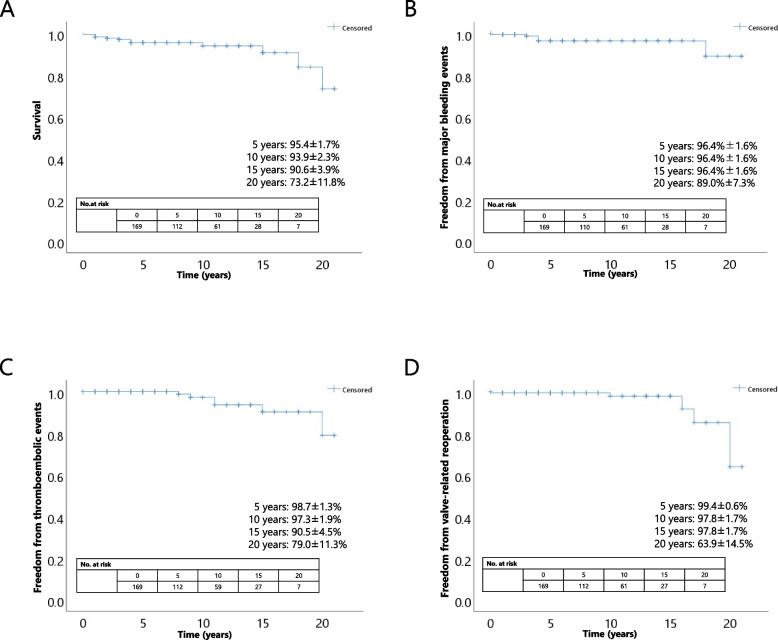
Kaplan–Meier curve. **a** Kaplan–Meier curve of freedom from survival. **b** Kaplan–Meier curve of freedom from major bleeding. **c** Kaplan–Meier curve of freedom from thromboembolic events. **d** Kaplan–Meier curve of freedom from valve-related reoperation

During the observation period, 10 late deaths occurred, including 7 cardiac-related deaths. The causes of late death are summarized in Table 3. The overall survival rate was 95.4 ± 1.7% at 5 years, 93.9 ± 2.3% at 10 years, 90.6 ± 3.8% at 15 years, and 73.2 ± 11.8% at 20 years (Fig. 2a). The results of Cox proportional hazards regression analysis for mortality after surgery is shown in Table 4. Hemodialysis was a prognostic factor of mortality (HR 0.034; *P* =  < 0.001).

**Table 3:** Causes of late death

Variables	n (%)
Overall	10
Cardiac related	7 (70%)
Prosthetic valve endocarditis	2
Hemorrhage	3
Stroke	2
Sepsis	2 (20%)
Unknown	1 (10%)

**Table 4:** Cox proportional hazards regression analysis for mortality after surgery

Variables	Univariable	
	HR (95% Cl)	*P*-value
<40 (years)	0.832 (0.158-9.889)	0.832
50-59 (years)	0.934 (0.272-3.212)	0.914
HD	0.034 (0.008-0.152)	< 0.001
Bioprosthetic valve	0.974 (0.123-7.706)	0.980
BAV	7.136 (0.911-55.897)	0.061
Infective endocarditis	25.927 (0.016-41317.730)	0.387
Rheumatic	0.593 (0.128-2.754)	0.505
Aortitis	0.188 (0.019-1.819)	0.149
AAR	26.761 (0.025-28554.045)	0.356
MVR	1.149 (0.144-9.166)	0.896
CABG	0.351 (0.043-2.858)	0.328

*AAR* ascending aortic replacement, *BAV* bicuspid aortic valve, *CABG* coronary artery bypass grafting, *HD* hemodialysis, *MVR* mitral valve replacement

#### Major bleeding

In total, six major bleeding events occurred during the follow-up period, all of which occurred in patients with mechanical valves. Freedom from major bleeding was 96.4 ± 1.6% at 5, 10, and 15 years and 89.0 ± 7.3% at 20 years (Fig. 2b).

#### Thromboembolism

In total, six thromboembolic events occurred during the follow-up period, all of which occurred in patients with mechanical valves. Freedom from thromboembolic events was 98.7 ± 1.3% at 5 years, 97.3 ± 1.9% at 10 years, 90.5 ± 4.5% at 15 years, and 79.0 ± 11.3% at 20 years (Fig. 2c).

#### Reoperation

There were six valve-related reoperations during the follow-up period, two of which were performed in patients with a bioprosthetic valve (one case each of perivalvular leaks and SVD) and four in patients with a mechanical valve (two cases of pannus formations and two cases of endocarditis). The causes of valve-related reoperation are detailed in Table 5. Freedom from valve-related reoperation was 99.4 ± 0.6% at 5 years, 97.8 ± 1.7% at 10 and 15 years, and 63.9 ± 14.5% at 20 years (Fig. 2d).

**Table 5:** Details of valve-related reoperation

Patient	Age/sex	Time from initial surgery (y)	Valve type	Reason for redo-surgery	Details of redo-surgery
1	55/F	0	CEP 21 mm	Perivalvular leak	AVR (CEP 21 mm)
2	54/F	10	On-X 19 mm	Pannus formation	AVR (ATS 20 mm)
3	75/M	16	ATS 20 mm	Endocarditis	Bentall (Crown 23 mm) + MVR (Mosaic 31 mm)
4	70/M	17	ATS 22 mm	Pannus formation	AVR (INSPIRIS 25 mm)
5	52/M	20	Mosaic 23 mm	SVD	AVR (St Jude 23 mm)
6	68/M	20	ATS 22 mm	Endocarditis	AVR (Trifecta 25 mm)

ATS (Medtronic, Minneapolis, MN, USA), *AVR* aortic valve replacement, *CEP* Carpentier-Edwards Magna Ease (Edwards Lifesciences LLC, Irvine, CA, USA), Crown (Sorin Group, Burnaby, Canada), *F* female; INSPIRIS RESILIA (Edwards Lifesciences LLC), *M* male; Mosaic (Medtronic, Inc.), *MVR* mitral valve replacement, St. Jude; St. Jude Medical Inc, St. Paul, MN, USA, Trifecta (Abbott Vascular, Santa Clara, CA, USA)

## Discussion

This study demonstrated that the long-term outcomes of AVR in patients aged < 60 years were acceptable. The 15- and 20-year survival rates were 90.6 ± 3.8% and 73.2 ± 11.8%, respectively. Freedom from major bleeding events, valve-related reoperation, and thromboembolic events at 15 years was > 95%, 95%, and 90%, respectively.

Major bleeding remains the most devastating complication of AVR with mechanical valves. Reports comparing long-term outcomes after isolated AVR in patients aged 50–69 years are common, depending on whether they received a bioprosthetic or mechanical prosthetic valve [10–13]. Two retrospective studies of > 4000 patients aged 50–69 years reported a higher rate of major bleeding with a mechanical prosthesis [10, 11]. Few studies have compared the long-term outcomes of isolated AVR in patients aged < 60 years who underwent operation after the year 2000 [14, 15]. These studies reported that there was no difference between freedom from major bleeding and thromboembolic events at the 10-year follow-up in both groups in patients aged < 60 years [14, 15]. Wang et al. [15] reported that freedom from major bleeding events at 5 and 10 years was 98.1% and 96.9% in patients with bioprosthetic valves and 95.4% and 91.5% in patients with mechanical valves, respectively. Freedom from major bleeding events in this study was similar to that previously reported, although mechanical valve usage rate for AVR was 89.4% (152/170 patients).

The guidelines’ recommendation for anticoagulation of bileaflet mechanical valves in the aortic position is an international normalized ratio (INR) of 2.0–2.5 [16]. We infer that the lower incidence of major bleeding events in our study could be related to the strict INR control of 2.0–2.5. Additionally, the target INR was adjusted and controlled individually according to the risk of major bleeding or embolism. The Prospective Randomized On-X Valve Reduced Anticoagulation Clinical Trial (PROACT) reported that INR was safely maintained at 1.5–2.0 in high-risk patients, without differences in mortality or thromboembolic complications [17]. Recent data show that lower INR targets could reduce the rates of valve-related events.

With recent trends showing an increase in bioprosthetic valve use because of an active lifestyle and avoidance of lifelong anticoagulation [18], the risk of reoperation for SVD in patients with bioprosthetic valves has increased in younger patients [19]. Bourguignon et al. reported that freedom from SVD with bioprosthetic valve placement for patients aged < 60 years at 15 and 20 years was 66.8% and 37.2%, respectively. These patients also showed a higher risk of reoperation [20]. Conversely, Bouhout et al. reported that freedom from reoperation with a mechanical valve at 10 years was 94.1% in patients with a mean age of 53 years at the time of surgery [21]. In a propensity-matched cohort study by Christ et al. (year of operation: 1993–2002), freedom from reoperation with a mechanical valve after 20 years was 90.4% in patients aged < 60 years [22]. This study reported that 6 patients underwent reoperation. Five patients required valve-related reoperation more than 10 years after the initial surgery, four of which were mechanical valves: two for pannus formation and two for endocarditis. One patient with a bioprosthetic valve required reoperation for SVD 20 years after the initial surgery. Although reoperation after mechanical valve implantation is not commonly needed, prosthetic dysfunction remains a persistent concern. A recent annual report by the Japanese Association for Thoracic Surgery showed that the 30-day mortality for initial surgical AVR and redo-AVR was 1.9% and 3.4%, respectively [19]. Redo-AVR has approximately twice the surgical mortality rate as initial surgery. Fortunately, there have been no reports of mortality associated with redo-AVR.

The use of bioprosthetic valves has markedly increased in Japan [6]. Most patients who opt for prosthetic valves are informed about receiving or expect to receive ViV (SAVR in TAVR) in the future [23]. However, there are limited long-term data on ViV (SAVR in TAVR). A multicenter study with 1,006 ViV TAVR patients recently showed that these patients have an 8-year survival rate of only 38% [24]. Additionally, SAVR with a bioprosthetic valve may not be an optimal treatment choice unless there are no complications including additional treatment such as ViV for at least 15 years. Moreover, comparison with data such as that presented in this study is essential for the evaluation of long-term outcomes.

Our study has the inherent limitations of a single-institution retrospective study and was subject to selection bias. We could not compensate for the bias in patient selection by employing propensity matching to compare patients with similar backgrounds due to the small sample size.

Therefore, further multicenter clinical studies involving patients with similar backgrounds are warranted. Due to the small number of events, hemodialysis was a positive predictor through Cox proportional hazards regression analysis for mortality. Thus, long-term observation is warranted.

## Conclusion

We reported the long-term outcomes following AVR in 170 patients aged < 60 years. Contrary to the recent trend, a high proportion of mechanical valves were used; however, the early- and long-term outcomes were acceptable. Valve selection was deemed acceptable according to the recommendations proposed by the Japanese guidelines.

### Supplementary Information

**Additional file 1:** **Online Resource 1.** Mechanical valve: Valve types and sizes. S1) ATS (Medtronic, Minneapolis, MN, USA) in 91 patients, On-X (On-X Life Technologies Inc., Austin, TX, USA) in 40 patients, St. Jude (SJM) (St. Jude Medical Inc., St. Paul, MN, USA) in 24 patients, and CarboMedics (Sorin SpA, Milan, Italy) in 7 patients.

**Additional file 2:** **Online Resource 2.** Bioprosthetic valve: Valve types and sizes. S2) Carpentier-Edwards Magna Ease (Edwards Lifesciences LLC, Irvine, CA, USA) in 11 patients, INSPIRIS RESILIA (Edwards Lifesciences LLC) in 8 patients, Mosaic bioprosthesis (Medtronic, Inc., Minneapolis, MN, USA) in 2 patients, and Trifecta (Abbott Vascular, Santa Clara, CA, USA) in 1 patient.

## Data Availability

The data that support the findings of this study are available upon request. Due to privacy and confidentiality restrictions, the raw data cannot be publicly shared. However, summarized and anonymized data can be made available to researchers who meet the criteria for access.
