# Supplementary material for: Twenty-year experience following aortic valve replacement in patients younger than 60 years of age
Source: J Cardiothorac Surg. 2024 May 7;19:279. doi: 10.1186/s13019-024-02776-x (PMC11075206; doi:10.1186/s13019-024-02776-x)
Supplement: Supplementary file 1 — Additional file 1: Online Resource 1. Mechanical valve: Valve types and sizes. S1) ATS (Medtronic, Minneapolis, MN, USA) in 91 patients, On-X (On-X Life Technologies Inc., Austin, TX, USA) in 40 patients, St. Jude (SJM) (St. Jude Medical Inc., St. Paul, MN, USA) in 24 patients, and CarboMedics (Sorin SpA, Milan, Italy) in 7 patients. [file 13019_2024_2776_MOESM1_ESM.pptx]

## Slide 1
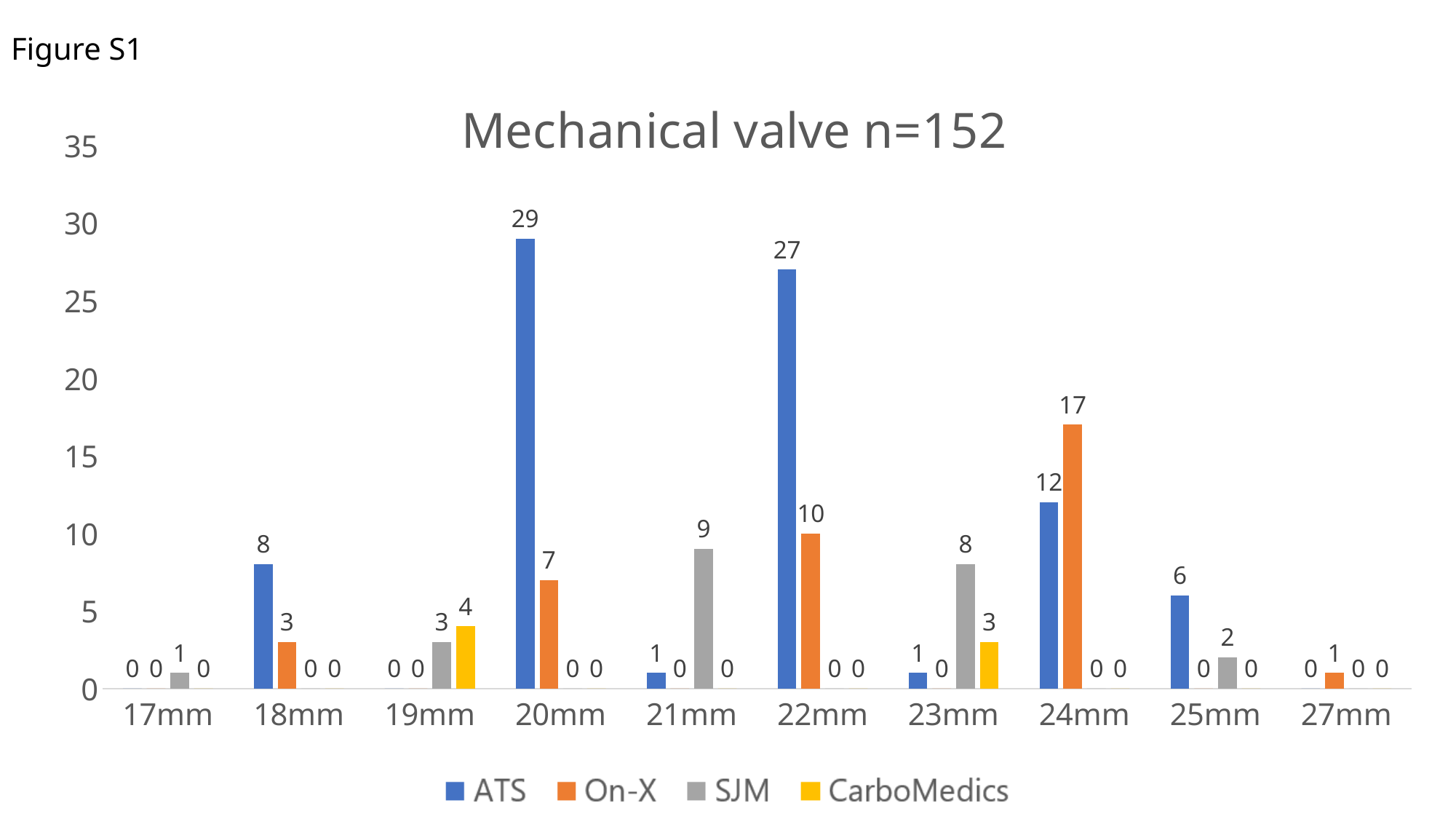

# Figure S1
### Chart: Mechanical valve n=152
| Category | ATS | ON-X | SJM | CarboMedics |
|---|---|---|---|---|
| 17mm | 0.0 | 0.0 | 1.0 | 0.0 |
| 18mm | 8.0 | 3.0 | 0.0 | 0.0 |
| 19mm | 0.0 | 0.0 | 3.0 | 4.0 |
| 20mm | 29.0 | 7.0 | 0.0 | 0.0 |
| 21mm | 1.0 | 0.0 | 9.0 | 0.0 |
| 22mm | 27.0 | 10.0 | 0.0 | 0.0 |
| 23mm | 1.0 | 0.0 | 8.0 | 3.0 |
| 24mm | 12.0 | 17.0 | 0.0 | 0.0 |
| 25mm | 6.0 | 0.0 | 2.0 | 0.0 |
| 27mm | 0.0 | 1.0 | 0.0 | 0.0 |
