# Supplementary figures and images for: Twenty-year experience following aortic valve replacement in patients younger than 60 years of age
Source: J Cardiothorac Surg. 2024 May 7;19:279. doi: 10.1186/s13019-024-02776-x (PMC11075206; doi:10.1186/s13019-024-02776-x)

## Slide 1
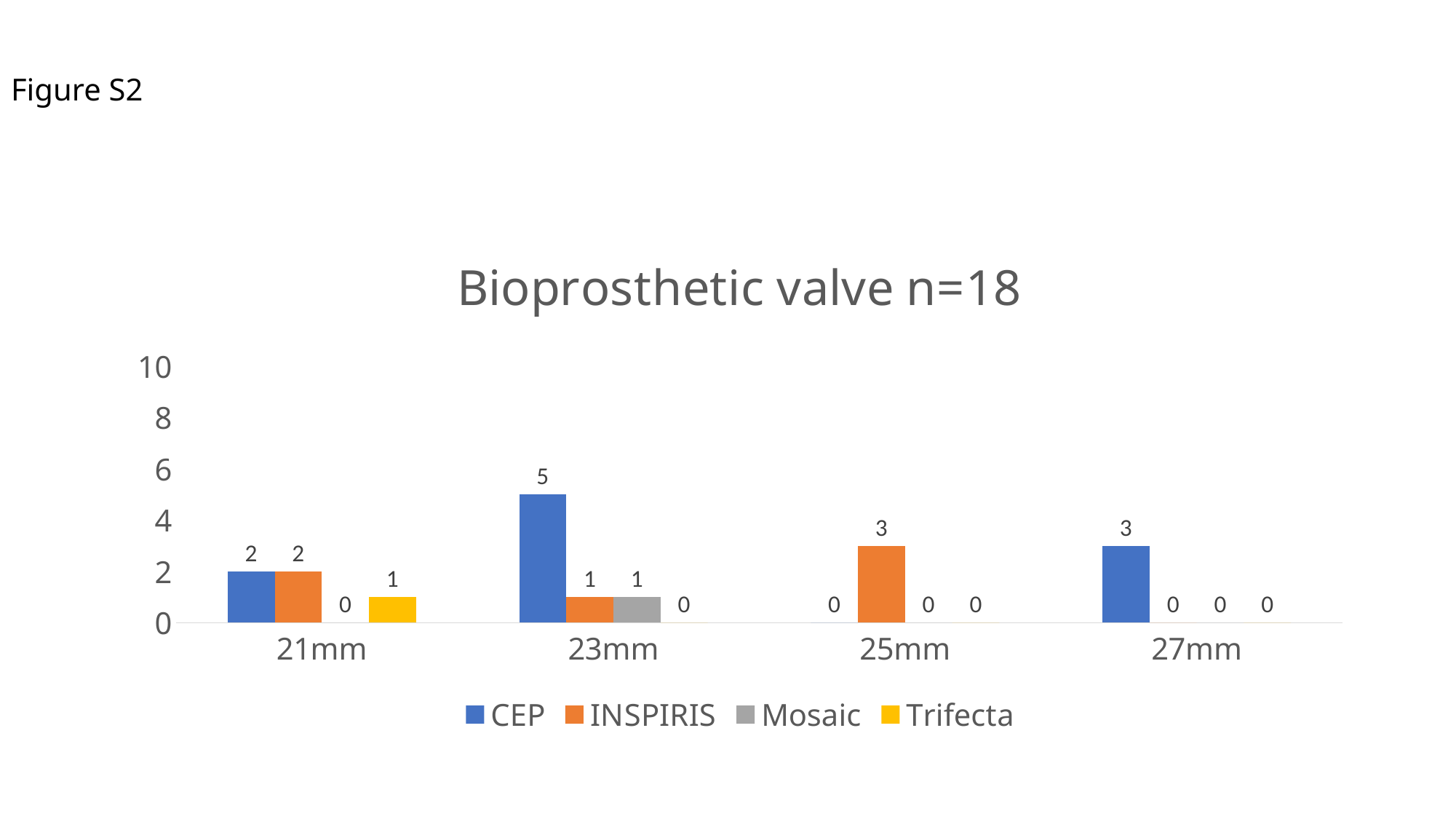

# Figure S2
### Chart: Bioprosthetic valve n=18
| Category | CEP | INSPIRIS | Mosaic | Trifecta |
|---|---|---|---|---|
| 21mm | 2.0 | 2.0 | 0.0 | 1.0 |
| 23mm | 5.0 | 1.0 | 1.0 | 0.0 |
| 25mm | 0.0 | 3.0 | 0.0 | 0.0 |
| 27mm | 3.0 | 0.0 | 0.0 | 0.0 |

Supplement: Supplementary file 2 — Additional file 2: Online Resource 2. Bioprosthetic valve: Valve types and sizes. S2) Carpentier-Edwards Magna Ease (Edwards Lifesciences LLC, Irvine, CA, USA) in 11 patients, INSPIRIS RESILIA (Edwards Lifesciences LLC) in 8 patients, Mosaic bioprosthesis (Medtronic, Inc., Minneapolis, MN, USA) in 2 patients, and Trifecta (Abbott Vascular, Santa Clara, CA, USA) in 1 patient. [file 13019_2024_2776_MOESM2_ESM.pptx]
